# Paradox of HIV stigma in an integrated chronic disease care in rural South Africa: Viewpoints of service users and providers

**DOI:** 10.1371/journal.pone.0236270

**Published:** 2020-07-31

**Authors:** Soter Ameh, Lucia D’Ambruoso, Francesc Xavier Gómez-Olivé, Kathleen Kahn, Stephen M. Tollman, Kerstin Klipstein-Grobusch

**Affiliations:** 1 Department of Community Medicine, Faculty of Medicine, University of Calabar, Calabar, Cross River State, Nigeria; 2 Medical Research Council/Wits University Rural Public Health and Health Transitions Research Unit (Agincourt), School of Public Health, Faculty of Health Sciences, University of the Witwatersrand, Johannesburg, South Africa; 3 Department of Global Health and Population, Harvard T.H. Chan School of Public Health, Boston, Massachusetts, United States of America; 4 Institute of Applied Health Sciences and Centre for Sustainable International Development, University of Aberdeen, Aberdeen, United Kingdom; 5 The International Network for the Demographic Evaluation of Populations and Their Health in Developing Countries (INDEPTH) Accra, Accra, Ghana; 6 Umea Centre for Global Health Research, Epidemiology and Global Health, Umea University, Umea, Sweden; 7 Division of Epidemiology and Biostatistics, School of Public Health, Faculty of Health Sciences, University of the Witwatersrand, Johannesburg, South Africa; 8 Julius Global Health, Julius Center for Health Sciences and Primary Care, University Medical Center Utrecht, Utrecht University, Utrecht, The Netherlands; Emory University School of Public Health, UNITED STATES

## Abstract

**Background:**

An integrated chronic disease management (ICDM) model was introduced by the National Department of Health in South Africa to tackle the dual burden of HIV/AIDS and non-communicable diseases. One of the aims of the ICDM model is to reduce HIV-related stigma. This paper describes the viewpoints of service users and providers on HIV stigma in an ICDM model in rural South Africa.

**Materials and methods:**

A content analysis of HIV stigmatisation in seven primary health care (PHC) facilities and their catchment communities was conducted in 2013 in the rural Agincourt sub-district, South Africa. Eight Focus Group Discussions were used to obtain data from 61 purposively selected participants who were 18 years and above. Seven In-Depth Interviews were conducted with the nurses-in-charge of the facilities. The transcripts were inductively analysed using MAXQDA 2018 qualitative software.

**Results:**

The emerging themes were HIV stigma, HIV testing and reproductive health-related concerns. Both service providers and users perceived implementation of the ICDM model may have led to reduced HIV stigma in the facilities. On the other hand, service users and providers thought HIV stigma increased in the communities because community members thought that home-based carers visited the homes of People living with HIV. Service users thought that routine HIV testing, intended for pregnant women, was linked with unwanted pregnancies among adolescents who wanted to use contraceptives but refused to take an HIV test as a precondition for receiving contraceptives.

**Conclusions:**

Although the ICDM model was perceived to have contributed to reducing HIV stigma in the health facilities, it was linked with stigma in the communities. This has implications for practice in the community component of the ICDM model in the study setting and elsewhere in South Africa.

## Background

The high dual burden of chronic non-communicable diseases (NCDs) and HIV/AIDS typifies South Africa’s epidemiological transition [1] which has implications for its health system. Globally, hypertension is the key risk factor for cardiovascular diseases [2] which in turn are the leading cause of mortality due to NCDs. It is estimated that 43% of South African adults are hypertensive [3] and nearly 50% of all deaths in South Africa are due to NCDs [4].

Similarly, the Human Immune-deficiency Virus (HIV) prevalence in South Africa was estimated at 13.1% in 2018, one of the highest in Africa [5]. Approximately 7.52 million people lived with HIV in 2018, with adults 15–49 years having a higher prevalence of 19% than the general population [5]. The HIV burden in South Africa is unequal by race, age and sex. The prevalence in black populations is 40–50 times that of white [6] and age-adjusted HIV prevalence differs significantly between women (26%) and men (19%) [7]. In adolescents, the risks of infection are eight times higher in females than males [6]. One of the main challenges People Living with HIV (PLWH) face is stigmatisation which is considered to occur at three levels: enacted, anticipated and internalized. Enacted stigma occurs when PLWH believe they experience prejudice and discrimination [8, 9]. Anticipated stigma happens when PLWH have an expectation that they will experience prejudice and discrimination [10]. Internalized stigma refers to a situation where PLWH endorse the negative beliefs and feelings linked with HIV/AIDS [9].

These forms of stigma are linked with negative health outcomes. Enacted and internalized stigma have been linked with increased prevalence of mental health disorders in different settings [11–13], particularly depression [14, 15] and anxiety [16, 17]. South African adults who experience high levels of internalized stigma have been reported to be less help-seeking and show signs of post-traumatic stress disorder [18]. Another study in South Africa showed that anticipated stigma was a barrier to care. Although PLWH reported less enacted stigma or hostility, they felt that HIV was synonymous with promiscuity and infidelity [19]. HIV stigmatisation could negatively impact achievement of the zero discrimination and 95-95-95 (i.e. 95% of people who live with HIV knowing their status, 95% of people who know their status receiving treatment and 95% of people on HIV treatment having a suppressed viral load) targets of ending the HIV/AIDS epidemic by 2030 in the context of the Sustainable Development Goals (SDGs) [20].

It is feasible to tackle the burden of HIV/AIDS and NCDs due to the commonalities in their prevention, management and control [21]. For instance, HIV and many NCDs have minimal or no symptoms at early stages of onset; thus, requiring a model of care different from acute care model. Next, both conditions require ongoing clinic appointments and adherence; hence, the need for approaches that entail appropriate appointment and medication reminder systems, adherence, self-management and referrals [22]. Furthermore, antiretroviral treatments (ARTs) increase the risk of lipidaemia and diabetes due to physiological mechanisms. This implies that PLWH and who are on ART have an increased chance of developing NCDs from three sources: first, from HIV infection itself; second, from ARTs; and third, from increasing age [23].

Early evidence showing the feasibility of integrating HIV/AIDS and NCD care emerged from a pilot study in Cambodia [24]. The findings showed that: 57% of diabetes patients had their glycosylated haemoglobin values equal to or less than the target of 9%, nearly 70% of hypertension patients on regular treatment for more than six months had their blood pressure values equal to or below the target of 160/90 mmHg and the median CD4 count of PLWH increased from 53 cells/mm^3^ at baseline to 316 cells/mm^3^ at the end of the second year of treatment. Furthermore, the same study showed that an integrated care for HIV/AIDS, hypertension and diabetes had a positive effect on HIV/AIDS-related stigma [24].

The pilot programme in Cambodia demonstrated the feasibility of providing an integrated care in the same clinic without HIV stigma being an obstacle to access to services. In view of the evidence in South Africa that HIV stigma is a barrier to care [19] and PLWH experience high levels of internalized stigma and are, therefore, less help-seeking [18], lessons learnt from the Cambodia study could serve as an impetus to reduce HIV stigma in South Africa.

To reduce HIV stigmatisation and improve patient health outcomes in South Africa [1, 25], the National Department of Health in 2011 introduced an Integrated Chronic Disease Management (ICDM) model as a pilot intervention programme in primary health care (PHC) facilities using the health systems approach [26, 27]. The purpose of the ICDM model of care is to leverage the HIV vertical programme to support or scale up services for NCDs to reduce HIV stigmatisation and to improve health outcomes of patients with NCDs [24]. The model has a facility component in which facilities are reorganised to enable providers to offer services for HIV and NCDs in a ‘one-stop-shop’ in designated chronic care areas. The community component of the model has a PHC outreach team comprising a nurse and community healthcare workers who visit patients’ homes to provide home-based care and link clinic defaulters back to care [28].

Other than the Cambodian study [24], other studies exploring integrated models of care have also been reported in Uganda [29], Malawi [30] and Kenya [31]. However, little is known about HIV stigmatisation in the context of the ICDM model of care using a qualitative inquiry. The aim of this research was to assess HIV stigma from the viewpoints of service users and providers in a rural setting in South Africa.

## Materials and methods

### Study site

The site for this study was in the Agincourt sub-district, Mpumalanga Province, South Africa. During commencement of data collection (November and December 2013), ICDM was being implemented in 17 of the 38 PHC facilities in the municipality. Seven of the 17 PHC facilities were selected because they are situated in the Ehlanzeni health district where the Medical Research Council/Wits Agincourt Research Unit has surveyed the population since 1992 using a Health and Demographic Surveillance System (HDSS). The population under surveillance in the Agincourt HDSS is 90,000 people in 16,000 households living in 27 villages [32].

### Study design and population

This is a content analysis of the ICDM model implemented in PHC facilities and their catchment communities. It was a part of a broader mixed methods research project which evaluated the quality of care in the ICDM model [33, 34] in the study setting. The study population consisted of patients 18 years and above being managed for HIV, hypertension or diabetes in the seven PHCs in the sub-district. The facility managers (i.e. professional nurses-in-charge of the seven facilities) were also considered part of the study population because of their viewpoints as service providers and facility managers.

### Inclusion and exclusion criteria

Patients diagnosed with and being managed in the health facilities for either HIV or hypertension or diabetes were eligible to participate in the study. Inclusion criteria were being on treatment six months before the ICDM model was implemented, participating in the health facility quantitative exit interviews on quality of the ICDM model of care commenced in June 2013 [33], overwhelmingly reporting (dis)satisfaction in the exit interviews and willingness to participate in the current Focus Group Discussions (FGDs). The quantitative survey, which preceded the qualitative study, was a cross-sectional survey of patients who were interviewed in the health facilities after consultations with the nurses (patient exit interviews). The survey was designed to contribute to understanding the quality of care in the ICDM model. The managers of the seven health facilities in the district were purposively selected for the In-depth Interviews (IDIs) based on their depth of experience as service providers and facility managers.

### Data collection

The quantitative exit interviews provided a large sampling frame from which purposive selection of prospective participants for the FGDs was done to explore patients’ in-depth perspectives on the dimensions of quality of care in the ICDM model. These dimensions of care were the pre-identified (a priori) topics in the qualitative component [34] of the boarder mixed methods research earlier described. During the exit interviews of the 435 randomly selected patients [33], which were held during the official clinic working hours (8.00 am—4.30 pm local time) from Monday to Friday, 80 patients who met the inclusion criteria described above were briefed about the purpose of the study and scheduled dates of the discussions and invited for the FGDs. Of these 80 purposively selected patients, 61 men and women participated in eight FGDs (i.e. one FGD per health facility and one FGD for clinic defaulters, defined in this study as HIV or hypertension or diabetes patients who missed three or more consecutive clinic appointments as was observed through the review of clinic records). Although stigma is a highly personal issue which could have been better discussed individually and with a wide range of people, HIV stigma can be and has been explored using FGDs in South Africa [19, 35], East Africa [36, 37] and the United States of America [38].

Two qualitative fieldworkers conducted the FGDs with 5–9 participants of similar age with each session lasting 60–90 minutes. Using the FGD guide written in English (S1 File), the more senior fieldworker moderated the discussions in Tsonga language by translating the guide from English to Tsonga during the discussions, while the second fieldworker audiotaped the discussions and made field notes during and after the FGDs.

Seven IDIs were conducted with the nurses-in-charge of the seven health facilities who were all females. The Principal Investigator (PI, S.A.) conducted and audiotaped the IDIs with the facility managers in English using the IDI guide (S2 File) which contained the same topics as the FGD guide. The IDIs were held in the offices of the facility managers during lunch break with an average duration of 30 minutes. The FGDs for service users and IDIs for facility managers were held concurrently. It was not possible to conduct FGDs for the facility managers at a convenient time and venue because of the nature of their busy work schedule.

### Quality assurance

The PI did a two-day training session with two senior qualitative field workers who had worked in the Agincourt HDSS for at least 10 years. The PI briefed field workers about the purpose of the study and discussions were held on how to administer the semi-structured topic guide and facilitate the discussions encouraging participation and balanced coherent discussion. The topic guides for the FGDs and IDIs were developed from the validated multi-scale Patient Satisfaction Questionnaire-18 (S3 File) which was developed by Ware et al. to assess dimensions of patient satisfaction with quality of care [39]. The interview guides were pretested in another community outside the study setting. The audio recordings of the FGDs were translated and transcribed into English by the two qualitative field workers. A third qualitative field worker, who was blinded to the other two field workers, validated the transcriptions by listening to two of the eight audiotapes and translating them into English. There were no major differences between the first and second transcriptions. A similar procedure was used to assure data quality for the IDIs.

Efforts were made to address trustworthiness of the data. Credibility of the data was enhanced by triangulating the data through administration of the same questions in the topic guides to different categories of participants: FGD guide to the participants and in-depth interview guide to the facility managers. For the purpose of transferability of the study findings, a description of the participants and the research process was provided to enable a reader transfer the findings of this research to their settings [e.g. context in which the research was carried out (PHC facilities implementing the ICDM model), its setting (rural), sample (61 participants), sample strategy (80 purposively sampled prospective participants), demographic and socio-economic characteristics (black rural male and female participants from a generally low socio-economic background), inclusion and exclusion criteria, interview procedure and topics and excerpts from the interview guide] [40]. Furthermore, prospective FGD participants of similar age were recruited from the seven PHC facilities during clinic opening hours on week days. The purpose of selecting participants of similar age was to enable in-depth exploration, of lived experiences with quality of care capitalising on group interactions. The FGDs were held on Saturdays at a convenient time for most of the prospective participants and in generally centrally located designated venues within the catchment communities served by the seven health facilities. The FGDs were held outside facilities to encourage participants to freely express and communicate their experiences without fear of intimidation or victimisation. Concerns of dependability and reliability of the data via coding and verification of inconsistent codes are presented under the Data Analysis sub-section below. Overall, eight FGDs and seven IDIs were conducted and more could not be done because data saturation was achieved, as was done elsewhere in South Africa where eight FGDs were conducted for 41 participants [35].

Although the transcripts were not returned to the participants for comments and/or corrections, the moderator of the FGDs and interviewer of the facility managers summarised the discussions and interviews to the participants who were requested to identify missing information or gaps.

### Data analysis

**Fig 1:**
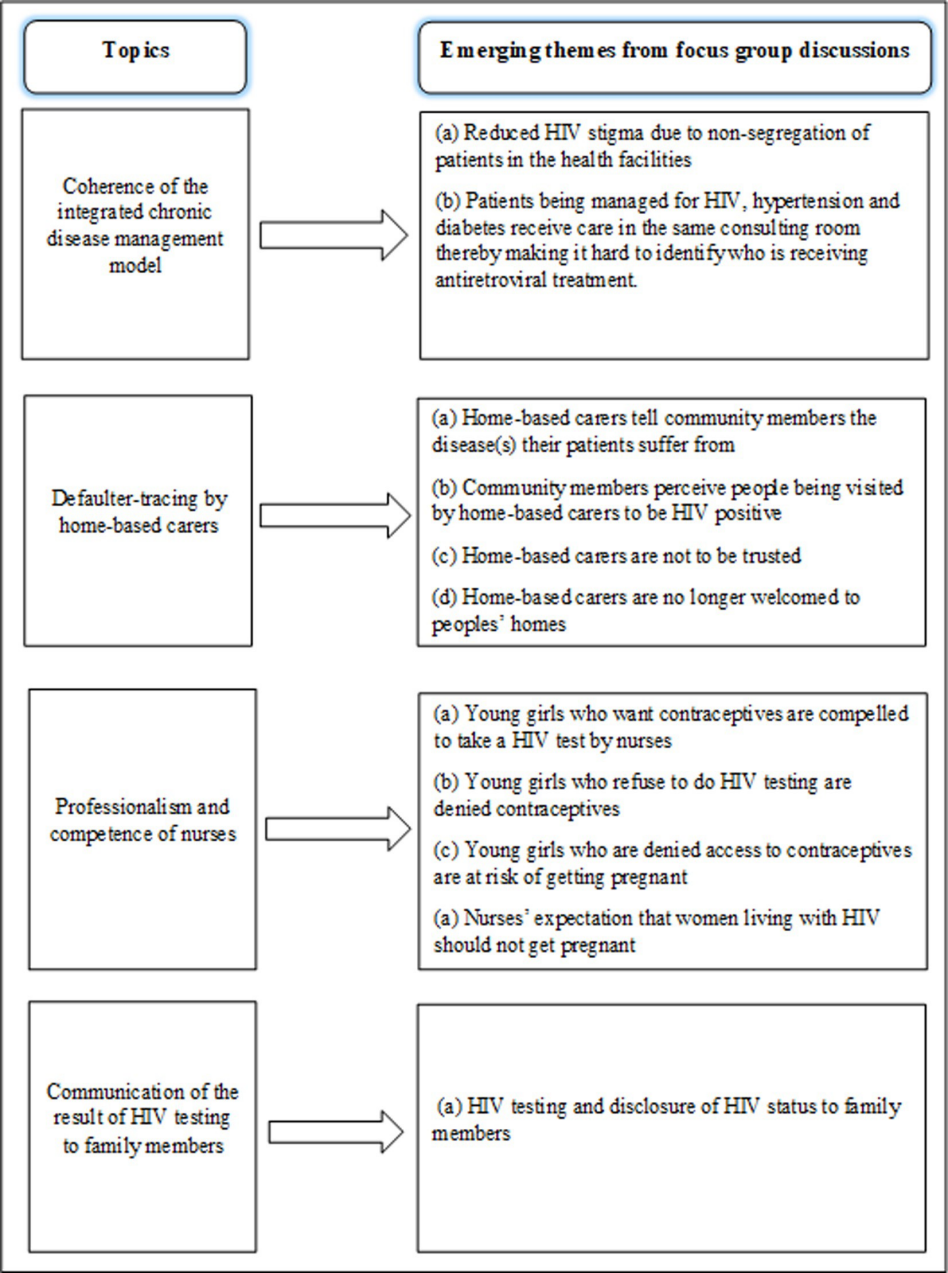
Schematic overview of the emerging themes from topics discussed in the focus groups and interviews.

**Fig 2:**
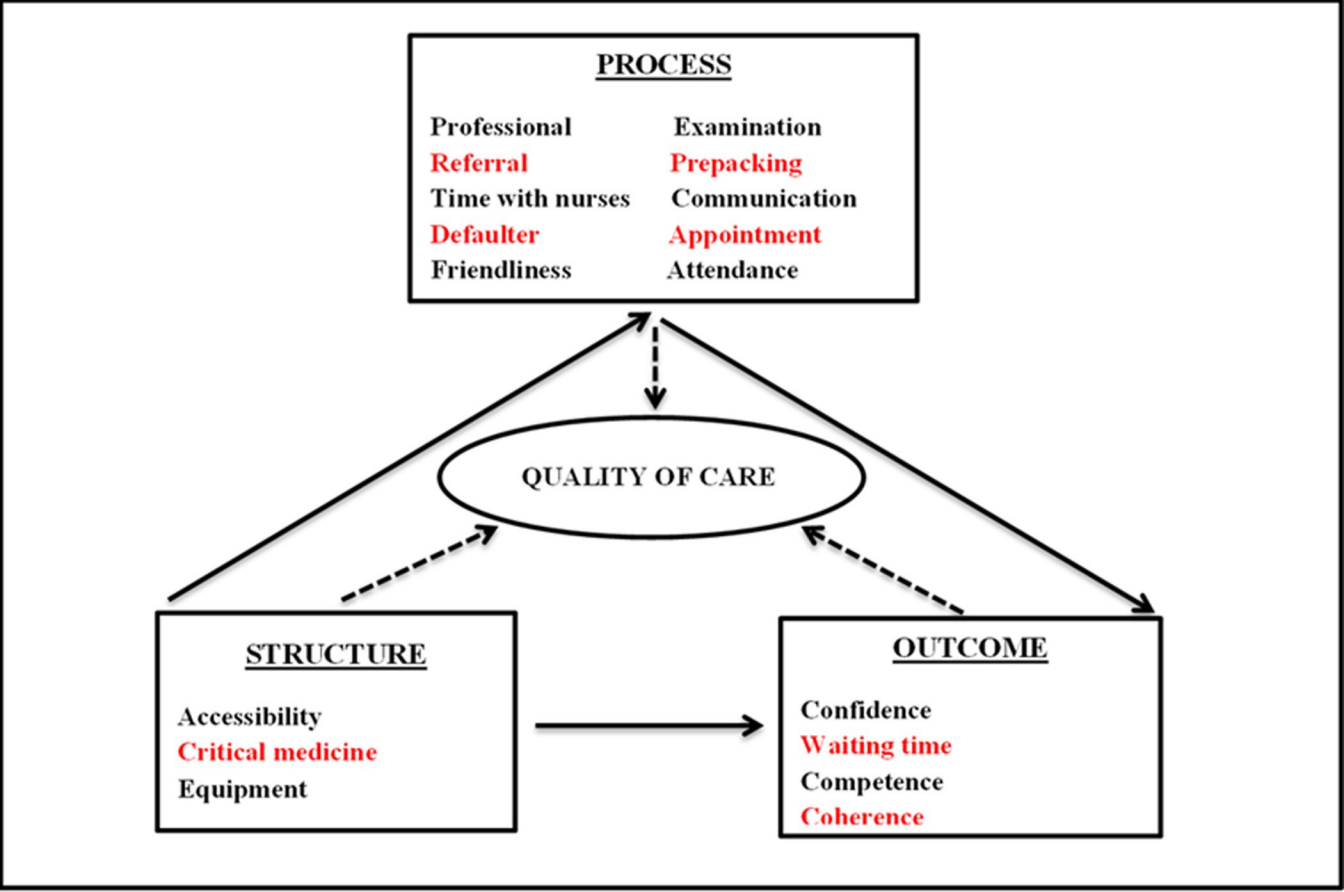
The 17 dimensions of quality of care and their intended structure, process and outcome constructs discussed in the focus groups and interviews in 2013. * The dimensions in red colour were the priority areas in the ICDM model.

The transcribed FGDs and IDIs were thematically analysed using MAXQDA 2018 qualitative software. The emerging theme on HIV stigma, HIV testing and reproductive health-related concerns were inductively analysed (i.e. the HIV stigma theme emerged as a new code not originally included in the coding framework). The analysis process began with multiple readings of the transcripts to allow themes unrelated to those in the topic guide to emerge. The PI coded the data through an iterative process and developed a codebook which was based on the recurring emerging themes. Interesting features in the texts were highlighted and dragged to the generated codes to reflect the meanings in the segments of the transcript. Each code and its texts were assigned the same colour to distinguish one code from another. Thereafter, some codes were grouped together in a hierarchical manner, like a coding tree, to form an overarching theme. The co-authors verified the codes through the reading and re-reading of the quotes, interrogated the data by discussing inconsistent codes and developed consensus on the emerging themes. This paper focuses on the emerging themes from the topics on quality of care in the ICDM model discussed in the FGDs and IDIs (Fig 1), while analysis of the 17 pre-identified (a priori) topics on quality of care (Fig 2) have been published elsewhere [34].

### Research team and reflexivity

The corresponding author and Principal Investigator (PI), SA, has over 20 years of experience in medical practice and 15 years of experience in conducting medical research. He attended organised courses on qualitative methods specifically to equip and prepare him to conduct this study. His published qualitative article on the 17 pre-identified themes on quality of care in the ICDM model was ranked 4^th^ among the 10 most cited articles in Health Policy and Planning Journal in 2019 (http://blogs.lshtm.ac.uk/hppdebated/2020/02/19/health-policy-and-plannings-top-10-articles-in-2019/).

The two senior qualitative fieldworkers were well known to the members of the communities where the study was conducted because they come from, live and work in these communities. Before the interviews and FGDs were conducted, the study team was introduced to the participants and the purpose of the study was explained to them. These details were in the information sheets which were given to the participants as their personal copies. One of the co-authors, LD, a female social scientist and a qualitative researcher at the Institute of Applied Health Sciences and Centre for Sustainable International Development University of Aberdeen, UK, supported the study team with technical expertise in the study design, conduct of the study, interpretation of the findings and drafting of the manuscript.

No biases or assumptions were reported about the interviewers. However, the interest of the research team and the purpose of the study was to get an in-depth perspective of health service users and providers on the quality of care in the ICDM model for the purpose of providing feedback to the managers of the ICDM model. After the study was conducted, a policy brief was submitted to the Mpumalanga Provincial Department of Health and the School of Public Health, University of the Witwatersrand, Johannesburg, South Africa.

All authors have a PhD degree: SA, FXGO, KK an ST are medical doctors, senior researchers, epidemiologists, public health scientists and university lecturers; LD is a social scientist, senior researcher and university lecturer; and KKG is an epidemiologist, a senior researcher and university lecturer.

The Agincourt Unit is a world class research centre which attracts researchers from academic institutions from all over the world. The unit has over twenty years of established relationship with the communities where the study participants live as well as the Provincial Department of Health who were aware of the study.

### Ethics statement

All procedures performed in studies involving human participants were in accordance with the ethical standards of the institutional and/or national research committee (University of the Witwatersrand, Johannesburg (M120943) and the Mpumalanga Provincial Research and Ethics Committee, South Africa.) and with the 1964 Helsinki declaration and its later amendments or comparable ethical standards. These Ethics Committees specifically approved this study. Written informed consent for the interviews/discussions and audio recordings were obtained from all individual participants in the study. These were countersigned by the interviewer or moderator.

### Data

The transcripts of the focus group discussions and in-depth interviews in this study contain sensitive information concerning people living with HIV as well as the clinics in which they accessed care. The Human Research Ethics Committee (Medical) of the University of the Witwatersrand, Johannesburg, South Africa imposes ethical restrictions on sharing the data for this study. However, data request may be sent to:

1. The Human Research Ethics Committee (Medical) of the University of the Witwatersrand, Johannesburg, South Africa. Telephone: +27-11-717-1234, Fax: +27117171265.
2. Medical Research Council/ Wits Rural Public Health and Health Transitions Research Unit (Agincourt), School of Public Health, Faculty of Health Sciences, University of the Witwatersrand, Johannesburg, South Africa. Telephone: +27-11-717-2085 and + 27-13-795-5076 (Acornhoek), Fax: + 27-13-795-5076.

## Results

The viewpoints of service users and providers on HIV stigma (main theme), HIV testing and reproductive health-related concerns (minor themes) are presented below. Verbatim quotes reflecting the viewpoints of women and their running commentaries have been used to illustrate the main theme on HIV stigma and the subthemes on enacted, anticipated and internalised domains of stigma. This was also applicable to the minor themes.

### Socio-demographic characteristics of the study population

The number of FGD participants by gender is shown in Table 1. There were a total of 61 participants: 43 females and 18 males. The higher number of female than male participants reflects a high female: male ratio of 7:3 in an earlier population-based study in the setting [41] as well as the facility-based study [33] from which the participants in this study were recruited. All the seven facility managers were females aged 40 to 55 years.

**Table 1:** Number of focus group discussion participants by gender.

Number of FGDs	Gender		Total
	Female	Male	
FGD 1	4	3	7
FGD 2	4	3	7
FGD 3	6	2	8
FGD 4	7	2	9
FGD 5	6	2	8
FGD 6	7	2	9
FGD 7	5	3	8
FGD for defaulters	4	1	5
Total	43	18	61

### Reduced HIV stigma in the health facilities

A facility manager, whose views represented that of many managers, reported that the ICDM model of care reduced HIV stigma by other patients due to non-segregation of patients managed for chronic diseases in the same clinic. This was because the former practice of segregating patients according to the illness they were being managed for made it easy to identify who was receiving treatment for HIV/AIDS in the health facilities. This was corroborated in the experience of service users where a woman described how she did not fear being identified as a patient attending a follow-up clinic to receive anti-retroviral treatment because professional nurses treated hypertension and diabetes patients as well as PLWH in the same consultation room. This view was expressed by participants from some other health facilities.

Previously we were grouping them [patients] according to their diseases, but now they are put together. Patients living with HIV/AIDS are satisfied because they are mixed with those who are having hypertension and diabetes [IDI with Manager, Health Facility 6].

In the past, there used to be a separate clinic for HIV patients. But now, all of us (referring to chronic disease patients) are getting our medication in one room. It is not easy for people [referring to other patients] to say I am HIV positive [Respondent 1 (woman), FGD Health Facility 5].

### Stigma in home-based care

Facility managers recounted how some community members stigmatised ill people who were visited in their homes by home-based carers (HBCs). These community members were said to have perceived persons visited by HBCs to have HIV/AIDS, and that these persons responded by not allowing HBCs to visit their homes because they thought that HBCs divulged their personal information to some community members.

I told them not to come to my house any more. When I tell them something I expect them to report it to their seniors not to tell the whole community [Respondent 5 (woman), FGD for Health Facility 2].

Home-based carers are not accepted. They [those being visited at home] are thinking that other people [community members] will think that they are HIV positive and that is why the HBCs are not allowed to visit them at home [IDI with Manager, Health Facility 1].

### Reproductive health-related stigmatisation by nurses

It was recounted in some FGDs how interactions between nurses and pregnant women living with HIV who visited the facility to utilise antenatal care services showed an expectation that women on ART should not get pregnant or should not desire to have children because of their HIV positive status.

I gave my file that I am using to take treatment [referring to ART] and the other file for pregnancy [referring to antenatal care] and she [referring to a nurse] says: are you pregnant again? She said it in a bad way. Then I asked her why she is talking like that and she said that a person like me is not supposed to get pregnant. Then I said to her: if I am HIV positive, does it mean I am not supposed to have children? I know my status and I know how to take care of myself. I didn’t feel okay when she ask me that why am I pregnant. It means when a person is positive she is not supposed to get pregnant? It means I am different from other people? [Respondent 6 (woman) FGD for Health Facility 3].

### Routine HIV testing as a barrier to contraceptive use and family planning

Respondents reported that young women who visited facilities for the purposes of receiving contraceptives or family planning were compelled to take an HIV test, even though the national contraception and family planning policy did not prescribe such practice. Young women who refused to take an HIV test were denied access to contraceptives or family planning; hence, routine HIV testing was experienced as a barrier to uptake of reproductive health services.

What I have observed is that, there are illnesses that you have to volunteer to be tested when you go to the clinic. But when young women go to the clinic for contraceptives, they are forced to test for HIV whether they like it or not. If you refuse to test you don’t get what you were there for [Respondent 3 (woman), FGD for Health Facility 1].

Some participants in the FGDs recounted how young girls who were denied access to contraceptives because they refused to be tested for HIV by nurses in the health facilities were at risk of getting pregnant.

At the clinic, when they go for contraceptives, they [nurses] don’t give them [patients] without testing for HIV. The young girls refuse to test and they will go home without getting the contraceptives. When you see lots of young women being pregnant, it is because they are afraid to test for HIV. [Respondent 3 (woman) FGD for Health Facility 2].

### HIV testing and disclosure of patients’ HIV status to family members

Respondents expressed views that HIV testing and disclosure of HIV status of a sick family member would enable parents or caregivers to provide care and support services to ensure adherence to medication. Furthermore, such disclosure could equip family members with the knowledge to take preventive measures from being infected with HIV.

You find a person [family member] being told by the nurse that she has HIV, but she [patient] will not tell the family that she is having HIV. She will get sicker and she will not tell you and you will go around to the clinics and hospital trying to find a cure for her meanwhile she knows what is killing her [Respondent 3 (woman) FGD for Health Facility 2].

I once went to the clinic with my cousin and she refused to be tested, I told her that she has to test whether she likes it or not, and the nurses said to me that I must not force her because it’s not according to the law. I told them that she is sick and I am the one to take care of her, how am I going to do it without knowing what is eating her. So we have to fight them until they get tested [Respondent 3 (woman) FGD for Health Facility 2].

## Discussion

The main study finding showed convergent viewpoints of service users and providers on the ICDM model being linked with reduced HIV stigma in the health facilities because patients managed for HIV and NCDs were mixed together in the waiting and consultation rooms. On the other hand, both service users and providers also had convergent perceptions of HIV stigmatisation in the communities which were related to the recounted practices of HBCs visiting the homes of people, some of who were PLWH, who missed their clinic appointments. Other findings, from the viewpoints of service users, suggested that nurses expected women on ART should not get pregnant because of their HIV status and conducted routine HIV testing of non-pregnant young women who wanted to use contraceptives; hence, unwanted pregnancy was an unintended consequence of non-use of contraceptives due to their refusal to take a routine HIV test. Finally, caregivers wanted HIV testing and/or disclosure of HIV status of sick family members which they perceived was an enabler for them to support PLWH to adhere to ART and to take preventive measures from being infected with HIV.

The pattern of HIV stigmatisation observed in this study is a paradox. On the one hand, the ICDM model was linked with HIV stigma reduction in PHC facilities in the study setting, as has been reported in Cambodia where HIV care was integrated with those for hypertension and diabetes in designated hospitals [24]. However, the model was related to HIV stigmatisation in the communities, an unintended consequence of the ICDM model of care, where HBCs traced persons managed for HIV or NCDs who defaulted from the clinics for the purposes of linking them back to care. During such visits, community members identified the homes visited by HBCs and perceived that the persons being visited were PLWH. This may imply that the design of the community-based component of the ICDM model may not have sufficiently addressed HIV stigmatisation at the community level. As shown elsewhere in South Africa [42], a HIV stigma-reduction community hub intervention was successful in initiating the onset of changes in a community through the PLWH and people living close to PLWH as community mobilisers active in the community hub to mobilise their own communities towards HIV stigma reduction through social change. Lessons learnt from this HIV stigma-reduction community “hub” intervention could be useful in mobilising and educating HBCs and community members towards HIV stigma reduction in the study setting.

Although the ICDM model of care was linked with reduced HIV stigma in the health facilities, the perspective of service users was that enacted stigma still occurred among pregnant HIV positive women who visited the antenatal clinic. These women felt stigmatised when nurses told them they were not supposed to get pregnant because of their HIV status, and this is expected. Although not in the context of the ICDM model, health worker-related stigma of this nature has been reported in the provision of reproductive health care in Ilorin, Nigeria, largely due to misperceptions [43]. On the other hand, a study in Lagos, Nigeria showed that health workers were supportive of women living with HIV who wished to have babies [44]. In Ghana, a study showed that a higher sexual and reproductive health stigma score was linked with a history of pregnancy among adolescents [45]. These evidence suggest that health education programmes should target health workers who offer reproductive health services to address misperceptions for the purpose of providing a holistic service delivery in the context of the ICDM model in South Africa and elsewhere. Aside stigma, the nurses may have genuinely intended for the pregnant women to have lower reproductive intention, which is linked with HIV positive status. This has been reported in a study that used National Demographic and Health Surveys to determine the effect of HIV status on fertility intention among women in nine sub-Saharan African countries. The study showed that women who were HIV positive, with knowledge of their status, had lower odds of wanting more children [46].

Anticipated and internalised stigmas were experienced by people in the communities when HBCs visited their homes which were identifiable by community members. This could have negative implications for activities of HBCs in implementing the community component of the ICDM model. Lessons learned from a home-based intervention in southwestern Kenya which led to enhanced couple HIV testing and counselling services and improved health outcomes of pregnant women living with HIV [47] could be relevant in addressing HIV status disclosure in the HBC programme in the ICDM model used in South Africa.

Routine HIV testing for adolescents and young women in the study setting was perceived to be a barrier to uptake of reproductive health services and contravened South Africa’s Children’s Act [48]. Section 134 of the Act facilitates adolescents’ access to contraception with the aim of preventing sexually active children ≥ 12 years from contracting sexually transmitted infections (including HIV) or falling pregnant [48]. Routine HIV testing, which is intended for pregnant women, has its origin in the guideline on HIV testing established by the World Health Organization (WHO) in 2004 [49]. The guideline recommends routine HIV testing for all pregnancy-related visits by service providers, especially in high HIV transmission areas with the intention of reducing mother-to-child HIV transmission. This study showed evidence suggestive of health workers’ misapplication of the WHO guideline, through refusal to provide contraceptives to adolescents who declined to take an HIV test, led to unintended consequences such as unwanted pregnancy.

The WHO guidelines for HIV testing and counselling for adolescents living with HIV recommend early and full disclosure of HIV status of children of school age as this improves ART adherence [50]. The findings of this study suggests that parents or guardians wanted routine HIV testing and/or disclosures of the HIV status of their adolescent wards when they were ill. Parents thought this would enable them to take preventive measures from being infected with HIV, provide care and ensure adherence to ART. This is corroborated in a study in South Africa that showed that early and full disclosure is strongly linked with improved adherence amongst ART-initiated adolescents 10–19 years of age; hence, disclosure may be an essential tool in improving adolescent adherence and reducing mortality and onwards transmission [51].

Although there has been changes in treatment guidelines and advances in model of care after the data were collected in 2013, the findings of this study could still be of relevance in recommending policy and practice related to HIV stigma in the context of the ongoing nationwide scale-up of the ICDM model. This is because the way HIV stigma is viewed and perceived by service users and facility managers in the ICDM model seven years afterward may not have changed significantly.

The main strength of this study was that the use of a qualitative inquiry to explore HIV stigma in the context of the ICDM model of care in South Africa was well suited for this research; hence, the transferability of the findings to other African countries implementing the ICDM model. Specifically, the knowledge generated from examining contextual factors that promote or reduce HIV stigmatisation could have implications for recommending policies and practices in the nationwide scale-up of the ICDM model. Furthermore, the viewpoints of service users and providers on HIV stigma enhanced the robustness of the data generated to fill the knowledge gap. Limitations do exist despite the aforementioned strengths of this study. The findings of this study may not be generalised to the sub-population of chronic disease patients receiving care in PHC facilities implementing the ICDM model elsewhere because the study participants were recruited purposively, a non-probability sampling technique. Next, this research was conducted in a rural setting and, therefore, its findings may not necessarily reflect similar contextual factors in PHC facilities in urban and semi-urban areas where the ICDM model is being implemented. Finally, the use of an inductive analytical approach only and non-stratification of the FGDs by sex posed a limitation in this study. Although discussions around HIV stigma was not anticipated at the time the study was designed, stratification of the FGDs by sex could have been more appropriate in providing a gender-sensitive environment to elicit further discussions on lived experiences related to gender-specific HIV stigma.

## Conclusions

Application of a comprehensive ICDM model was linked with reduced HIV stigma in the health facilities, but was linked to stigmatisation in the communities. Routine HIV testing, originally intended for pregnant women, of non-pregnant adolescents who desired to use contraceptives was related to unwanted pregnancies. Refusal to provide contraceptives to adolescents who declined to take an HIV test could have far reaching implications for adolescent-friendly reproductive health services. Caregivers of PLWH wanted routine HIV testing and/or disclosures of the HIV status of their family members for the purposes of protecting their own health and enhancing adherence to ART.

## Supporting information

S1 File Focus group discussion guide. (PDF)

S2 File In-depth interview guide. (PDF)

S3 File Patient satisfaction questionnaire-18 used to develop the focus group and interview guides. (PDF)

## References

[1] Mayosi BM, Flisher AJ, Lalloo UG, Sitas F, Tollman SM, Bradshaw D. The burden of non-communicable diseases in South Africa. Lancet. 2009;374(9693):934–947. 10.1016/S0140-6736(09)61087-4 19709736

[2] Lim SS, Vos T, Flaxman AD, Danaei G, Shibuya K, Adair-Rohani H, et al A comparative risk assessment of burden of disease and injury attributable to 67 risk factors and risk factor clusters in 21 regions, 1990–2010: a systematic analysis for the Global Burden of Disease Study 2010. Lancet. 2012;380(9859):2224–2260. 10.1016/S0140-6736(12)61766-8 23245609 PMC4156511

[3] Ware LJ, Chidumwa G, Charlton K, Schutte AE, Kowal P. Predictors of hypertension awareness, treatment and control in South Africa: results from the WHO-SAGE population survey (Wave 2). J Hum Hypertens. 2019;33(2):157–166. 10.1038/s41371-018-0125-3 30382179

[4] World Health Organization (Geneva). Non-communicable diseases country profile 2014. Available from: http://www.who.int/nmh/countries/zaf_en.pdf?ua=1.

[5] Statistics South Africa. 2018. Mid-year population estimates. Available from: https://www.statssa.gov.za/publications/P0302/P03022018.pdf.

[6] Zuma K, Shisana O, Rehle TM, Simbayi LC, Jooste S, Zungu N, et al New insights into HIV epidemic in South Africa: key findings from the National HIV Prevalence, Incidence and Behaviour Survey, 2012. Afr J AIDS Res. 2016;15(1):67–75. 10.2989/16085906.2016.1153491 27002359

[7] Clark SJ, Gomez-Olive FX, Houle B, Thorogood M, Klipstein-Grobusch K, Angotti N, et al Cardiometabolic disease risk and HIV status in rural South Africa: establishing a baseline. BMC Public Health. 2015;15:135 10.1186/s12889-015-1467-1 25885455 PMC4335669

[8] Valenzuela C, Ugarte-Gil C, Paz J, Echevarria J, Gotuzzo E, Vermund SH, et al HIV stigma as a barrier to retention in HIV care at a general hospital in Lima, Peru: a case-control study. AIDS Behav. 2015;19(2):235–245. 10.1007/s10461-014-0908-7 25269871 PMC4344383

[9] Sorsdahl KR, Mall S, Stein DJ, Joska JA. The prevalence and predictors of stigma amongst people living with HIV/AIDS in the Western Province. AIDS Care. 2011;23(6):680–685. 10.1080/09540121.2010.525621 21360358

[10] Kingori C, Reece M, Obeng S, Murray M, Shacham E, Dodge B, et al Psychometric evaluation of a cross-culturally adapted felt stigma questionnaire among people living with HIV in Kenya. AIDS Patient Care STDS. 2013;27(8):481–488. 10.1089/apc.2012.0403 23968206

[11] Yi S, Chhoun P, Suong S, Thin K, Brody C, Tuot S. AIDS-related stigma and mental disorders among people living with HIV: a cross-sectional study in Cambodia. PloS One. 2015;10(3):e0121461–e0121461. 10.1371/journal.pone.0121461 25806534 PMC4373790

[12] Dow DE, Turner EL, Shayo AM, Mmbaga B, Cunningham CK, O'Donnell K. Evaluating mental health difficulties and associated outcomes among HIV-positive adolescents in Tanzania. AIDS care. 2016;28(7):825–833. 10.1080/09540121.2016.1139043 26837437 PMC4905805

[13] Deribew A, Tesfaye M, Hailmichael Y, Apers L, Abebe G, Duchateau L, et al Common mental disorders in TB/HIV co-infected patients in Ethiopia. BMC infect. Dis. 2010;10:201 10.1186/1471-2334-10-201 20618942 PMC2911449

[14] Endeshaw M, Walson J, Rawlins S, Dessie A, Alemu S, Andrews N, et al Stigma in Ethiopia: association with depressive symptoms in people with HIV. AIDS Care. 2014;26(8):935–939. 10.1080/09540121.2013.869537 24382290

[15] Kalomo EN. Associations between HIV-related stigma, self-esteem, social support, and depressive symptoms in Namibia. Aging Ment Health. 2018;22(12):1570–1576. 10.1080/13607863.2017.1387763 29019412

[16] Liu Y, Gong H, Yang G, Yan J. Perceived stigma, mental health and unsafe sexual behaviors of people living with HIV/AIDS. Zhong Nan Da Xue Xue Bao Yi Xue Ban 2014;39(7):658–663. 10.11817/j.issn.1672-7347.2014.07.002 25080902

[17] Tesfaw G, Ayano G, Awoke T, Assefa D, Birhanu Z, Miheretie G, et al Prevalence and correlates of depression and anxiety among patients with HIV on-follow up at Alert Hospital, Addis Ababa, Ethiopia. BMC Psychiatry. 2016;16(1):368 10.1186/s12888-016-1037-9 27806711 PMC5094082

[18] Breet E, Kagee A, Seedat S. HIV-related stigma and symptoms of post-traumatic stress disorder and depression in HIV-infected individuals: does social support play a mediating or moderating role? AIDS Care. 2014;26(8):947–951. 10.1080/09540121.2014.901486 24666226

[19] Treves-Kagan S, Steward WT, Ntswane L, Haller R, Gilvydis JM, Gulati H, et al Why increasing availability of ART is not enough: a rapid, community-based study on how HIV-related stigma impacts engagement to care in rural South Africa. BMC Public Health. 2015;16:87. 10.1186/s12889-016-2753-2 PMC4730651 26823077

[20] United Nations (New York). Sustainable Development Goals. Available from: https://www.un.org/sustainabledevelopment/health/.

[21] Levitt NS, Steyn K, Dave J, Bradshaw D. Chronic noncommunicable diseases and HIV-AIDS on a collision course: relevance for health care delivery, particularly in low-resource settings—insights from South Africa. Am J Clin Nutr. 2011;94(6):1690S–1696S. 10.3945/ajcn.111.019075 22089433 PMC3226022

[22] Joint United Nations Programme on HIV and AIDS (New York). Chronic care of HIV and non-communicable diseases: How to leverage the HIV experience. Available from: http://www.unaids.org/en/media/unaids/contentassets/documents/unaidspublication/2011/20110526_JC2145_Chronic_care_of_HIV-1.pdf.

[23] Smart T. HIV and non-communicable diseases (NCDs). 2011. http://www.aidsmap.com/HIV-and-non-communicable-diseases-NCDs/page/2094965/. Accessed March 13, 2012

[24] Janssens B, Van Damme W, Raleigh B, Gupta J, Khem S, Soy Ty K, et al Offering integrated care for HIV/AIDS, diabetes and hypertension within chronic disease clinics in Cambodia. Bull World Health Organ. 2007;85(11):880–885. 10.2471/blt.06.036574 18038079 PMC2636263

[25] Tollman SM, Kahn K, Sartorius B, Collinson MA, Clark SJ. Implications of mortality transition for primary health care in rural South Africa: a population-based surveillance study. Lancet. 2008;372 10.1016/S0140-6736(08)61155-1 18790312 PMC2602585

[26] Mahomed OH, Asmall S. Development and implementation of an integrated chronic disease model in South Africa: lessons in the management of change through improving the quality of clinical practice. International Journal of Integrated Care. 2015;15:e038 10.5334/ijic.1454 26528101 PMC4628546

[27] Mahomed OH, Asmall S, Freeman M. An integrated chronic disease management model: a diagonal approach to health system strengthening in South Africa. J Health Care Poor Underserved. 2014;25(4):1723–1729. 10.1353/hpu.2014.0176 25418238

[28] Mahomed OH, Asmall S, Voce A. Sustainability of the integrated chronic disease management model at primary care clinics in South Africa. African Journal of Primary Health Care & Family Medicine. 2016;8(1):1248. 10.4102/phcfm.v8i1.1248 PMC5125260 28155314

[29] Kwarisiima D, Atukunda M, Owaraganise A, Chamie G, Clark T, Kabami J, et al Hypertension control in integrated HIV and chronic disease clinics in Uganda in the SEARCH study. BMC Public Health. 2019;19(1):511 10.1186/s12889-019-6838-6 31060545 PMC6501396

[30] Patel P, Speight C, Maida A, Loustalot F, Giles D, Phiri S, et al Integrating HIV and hypertension management in low-resource settings: Lessons from Malawi. PLoS Medicine. 2018;15(3):e1002523–e1002523. 10.1371/journal.pmed.1002523 29513674 PMC5841643

[31] Venables E, Edwards JK, Baert S, Etienne W, Khabala K, Bygrave H. "They just come, pick and go." The Acceptability of Integrated Medication Adherence Clubs for HIV and Non Communicable Disease (NCD) Patients in Kibera, Kenya. PloS One. 2016;11(10):e0164634–e0164634. 10.1371/journal.pone.0164634 27764128 PMC5072644

[32] Kahn K, Collinson MA, Gomez-Olive FX, Mokoena O, Twine R, Mee P, et al Profile: Agincourt health and socio-demographic surveillance system. Int J Epidemiol. 2012;41(4):988–1001. 10.1093/ije/dys115 22933647 PMC3429877

[33] Ameh S, Gómez-Olivé FX, Kahn K, Tollman SM, Klipstein-Grobusch K. Relationships between structure, process and outcome to assess quality of integrated chronic disease management in a rural South African setting: applying a structural equation model. BMC Health Serv Res. 2017;17(1):229 10.1186/s12913-017-2177-4 28330486 PMC5363044

[34] Ameh S, Klipstein-Grobusch K, D’Ambruoso L, Kahn K, Tollman SM, Gómez-Olivé FX. Quality of integrated chronic disease care in rural South Africa: user and provider perspectives. Health Policy Plan. 2017; 32(2): 257–266. 10.1093/heapol/czw118 28207046 PMC5400067

[35] Bogart LM, Chetty S, Giddy J, Sypek A, Sticklor L, Walensky RP, et al Barriers to care among people living with HIV in South Africa: contrasts between patient and healthcare provider perspectives. AIDS Care. 2013;25(7):843–853. 10.1080/09540121.2012.729808 23061894 PMC3552028

[36] McHenry MS, Nyandiko WM, Scanlon ML, Fischer LJ, McAteer CI, Aluoch J, et al HIV Stigma: Perspectives from Kenyan Child Caregivers and Adolescents Living with HIV. Journal of the International Association of Providers of AIDS Care. 2017;16(3):215–225. 10.1177/2325957416668995 27655835 PMC5464367

[37] Duff P, Kipp W, Wild TC, Rubaale T, Okech-Ojony J. Barriers to accessing highly active antiretroviral therapy by HIV-positive women attending an antenatal clinic in a regional hospital in western Uganda. J Int AIDS Soc. 2010;13:37 10.1186/1758-2652-13-37 20863399 PMC2954932

[38] Rintamaki L, Kosenko K, Hogan T, Scott AM, Dobmeier C, Tingue E, et al The Role of Stigma Management in HIV Treatment Adherence. Int J Environ Res Public Health. 2019;16(24):5003. 10.3390/ijerph16245003 PMC6950713 31835334

[39] Ware JE, Snyder MK, Wright WR. Development and validation of scales to measure patient satisfaction with health care services: review of literature, overview of methods and results regarding construction of scales. Springfield: National Technical Information Service; 1976.

[40] Korstjens I, Mosner A. Series: Practical guidance to qualitative research. Part 4: Trustworthiness and publishing. Eur J Gen Pract. 2017; 23(1):1–2. 10.1080/13814788.2017.1291906 29202616 PMC8816392

[41] Ameh S, Gomez-Olive FX, Kahn K, Tollman SM, Klipstein-Grobusch K. Predictors of health care use by adults 50 years and over in a rural South African setting. Glob Health Action. 2014;7:24771 10.3402/gha.v7.24771 25087686 PMC4119936

[42] Prinsloo CD, Greeff M, Kruger A, Khumalo IP. HIV stigma experiences and stigmatisation before and after a HIV stigma-reduction community "hub" intervention. Afr J AIDS Res. 2017;16(3):203–213. 10.2989/16085906.2017.1349683 28978287

[43] Sekoni OO, Owoaje ET. HIV/AIDS stigma among primary health care workers in Ilorin, Nigeria. Afr J Med Med Sci. 2013;42(1):47–57. 23909094

[44] Ehiri JE, Alaofè HS, Yesufu V, Balogun M, Iwelunmor J, Kram NAZ, et al AIDS-related stigmatisation in the healthcare setting: a study of primary healthcare centres that provide services for prevention of mother-to-child transmission of HIV in Lagos, Nigeria. BMJ open. 2019;9(5):e026322–e026322. 10.1136/bmjopen-2018-026322 31110094 PMC6530297

[45] Hall KS, Morhe E, Manu A, Harris LH, Ela E, Loll D, et al Factors associated with sexual and reproductive health stigma among adolescent girls in Ghana. PloS One. 2018;13(4):e0195163–e0195163. 10.1371/journal.pone.0195163 29608595 PMC5880390

[46] Mumah JN, Ziraba AK, Sidze EM. Effect of HIV status on fertility intention and contraceptive use among women in nine sub-Saharan African countries: evidence from Demographic and Health Surveys. Glob Health Action. 2014;7:25579 10.3402/gha.v7.25579 25361729 PMC4212081

[47] Turan JM, Darbes LA, Musoke PL, Kwena Z, Rogers AJ, Hatcher AM, et al Development and Piloting of a Home-Based Couples Intervention During Pregnancy and Postpartum in Southwestern Kenya. AIDS patient care and STDs. 2018;32(3):92–103. 10.1089/apc.2017.0285 29620927 PMC5865625

[48] Department of Health, Republic of South Africa. National Contraception and Fertility Planning Policy and Service Delivery Guidelines. A companion to the National Contraception Clinical Guidelines, 2012. Available from: http://www.partners-popdev.org/wp-content/uploads/2015/08/National-contraception-family-planning-policy.pdf.

[49] The Joint United Nations Programme on HIV/AIDS /World Health Organization. UNAIDS/WHO Policy Statement on HIV Testing (2004). Available from: https://www.who.int/hiv/pub/vct/statement/en/.

[50] World Health Organization (Geneva). HIV and adolescents: Guidance for HIV testing and counselling and care for adolescents living with HIV 2013. Available from: https://apps.who.int/iris/bitstream/handle/10665/94334/9789241506168_eng.pdf?sequence=. 25032477

[51] Cluver LD, Hodes RJ, Toska E, Kidia KK, Orkin FM, Sherr L, et al 'HIV is like a tsotsi. ARVs are your guns': associations between HIV-disclosure and adherence to antiretroviral treatment among adolescents in South Africa. AIDS. 2015;29 (l):S57–65. 26049539 10.1097/QAD.0000000000000695

