# Supplementary material for: Paradox of HIV stigma in an integrated chronic disease care in rural South Africa: Viewpoints of service users and providers
Source: PLoS One. 2020 Jul 31;15(7):e0236270. doi: 10.1371/journal.pone.0236270 (PMC7394420; doi:10.1371/journal.pone.0236270)
Supplement: S1 File — (PDF) [file pone.0236270.s001.pdf]

VUNENE STUDY – FOCUS GROUP DISCUSSION GUIDE

TOPIC GUIDE FOR PARTICIPANTS

1. **INTRODUCTION**
   - Introduce members of the team
   - The aim of this discussion is to understand patient experiences with the quality of care for chronic diseases in the primary health facilities
   - We would like to get a very good understanding of your experiences in the clinics, some of which you may not have been able to discuss with us during interviews in the clinic.
   - This discussion will contribute in understanding how services for chronic diseases are organized in the health facilities
   - This study has the support of the Bushbuckridge Department of Health and Wits University
2. **ACTIVITIES**
   - Eleven topics around quality of chronic disease care in the clinic will be discussed today
   - Participation in this discussion will be free, fair, equal and in relaxed environment
   - A report of this discussion will be sent to you
   - There will be no mention of your names in the report or any other document(s)
   - You will not be identified as a participant in this discussion
   - Refreshments will be provided in the meetings
   - You are free to leave the study at any time
   - We would like to know if there are suggested modifications to this programme
3. **WRITTEN INFORMED CONSENT**
   - Written informed consent, signed by participants, study investigator or senior qualitative field worker is obtained

INTRODUCTION

Chronic diseases are diseases (e.g. hypertension, diabetes and HIV) that require regular and on-going, usually for a long time contact with health facilities. Since June 2011, the National Department of Health has been testing the integrated chronic disease management (ICDM) model of care in all the clinics Bushbuckridge sub-district. The aim of this model is to improve health outcomes of patients with chronic diseases and improve service delivery in health facilities.

We invite you to describe your experiences with the quality of care in the clinics with respect to the topics below. **You are not expected to name the chronic disease for which you are being managed in the clinic.**

Topics for the discussion

1) General satisfaction with the quality of the integrated chronic disease care

- In general, could you tell us how satisfied or dissatisfied you are with the integrated chronic disease care in the health facility you attend?
  - Prompt participants to explain the reason for their satisfaction or dissatisfaction
- All of you have been receiving treatment in the clinics for at least two years. Have you noticed any changes in the way services for chronic diseases have been combined?
  - Prompt participants to discuss any positive or negative changes in the combined/integrated chronic disease services in the clinics since June 2011
- How perfect are these services?
  - Prompt participants to consider what works and does not work from their point of view and invite them to explain why they feel this way
  - Invite participants to describe specific examples

2) Technical quality of care and confidence in the nurses

- What are your views on the clinical skills of the nurses who treat chronic diseases?
  - Do you wonder if their diagnosis is correct
  - Do you have doubts about the clinical skills of the nurses
  - Invite participants to illustrate their opinions and describe specific instances

3) Interpersonal relations and friendliness

- Tell us whether the nurses are very friendly and polite to you
  - Invite participants to explain why they agree or disagree with the above question

4) Professionalism and competence of nurses

- What do you think about the professional conduct of the nurses?
  - Probe participants to narrate specific instances of professional misconduct of the nurses
- What do you think about the competences of the nurses to manage chronic diseases?
  - Probe participants to describe specific instances of competence or incompetence of the nurses

5) Communication

- Tell us whether the nurses explain to you reason(s) for doing physical examination or requesting laboratory tests
  - Invite participants to describe such experiences
- Do the nurses sometimes ignore what you tell them?
  - Probe participants to provide illustrations with specific instances

6) Financial aspect of accessing care

- Do have to pay fees to access services in the clinics?
  - Invite participants to illustrate specific instances when fees were paid
- Are you able to afford the cost of transportation to the clinic?
  - Invite participants to describe distances between their homes and the clinic, and how much they have to pay for transport

7) Waiting time before seeing nurses and time spent with nurses in the consultation room

- How long do you wait to see the nurses before they attend to you?
  - Probe participants to describe their experiences with waiting time
- Do the nurses hurry too much when they attend to you in the consultation room?
  - Probe participants to describe their experiences

8) Accessibility of the integrated chronic disease services

- How hard or easy is it for you to access services in the clinic?
  - Invite participates to describe their experiences
- Describe your experiences in having access to a doctor or a specialist

9) Coherence of the integrated chronic disease services

- How well combined are the HIV, hypertension and diabetes services
  - Invite participants to describe their experiences

10) Referral

- Tell us whether the nurses sometimes refer you to the doctor or hospital when necessary
  - Invite participants to describe their experiences with referral.
  - Probe about back-referral from the doctors to the nurses in instances where participants were referred to the doctors by the nurses
  - Tell us your experiences when you were transferred from one clinic to another

11) Defaulter-tracing

- Tell us if community health workers visit the homes of people (HIV, hypertension and diabetes patients) who missed three consecutive clinic appointment
  - Explain to the discussants that the period for discussion is from 2013 to 2014
  - Probe participants whether the community (volunteer) health workers visited the homes of people when they missed their clinic appointments
  - Probe to ascertain if participants are referred back to the clinics by the community (volunteer) health workers

12) Supply of critical medicines

- Can you tell us about regular supply of critical medicines (antiretroviral drugs, antihypertensive medicines and anti-diabetes medicines) for the treatment of chronic diseases in the clinics?
  - Invite participants to describe their experiences with drug stock-outs.
  - What was the response of the nurses in such instances? (E.g. do nurses order drugs from neighbouring clinics in such instances of drug stock-outs?)

13) Equipment

- Can you tell us about adequacy and functionality of equipment in the clinics?
  - Invite participants to share their experiences on availability of equipment in the clinics (e.g. BP machines. Glucometers, weighing scale etc.).

    - Probe participants to ascertain instances when equipment was needed to provide care, but was not available
    - Invite participants to describe the functionality of equipment in the clinics (e.g. BP machines. Glucometers, weighing scale etc.).
    - How do the nurses cope in such instances when the equipment are broken?

14) Appointment system

- Can you tell us about the clinic appointment system?
- How hard is it to get an appointment for medical care as soon as you need it?
    - Invite participants to describe their specific experiences
    - What are the intervals of clinic appointment?
    - How do the nurses prescribe medications to last the period of clinic appointment?

15) Prepacking of medicines

- Do the nurses pack your medicines the day before your clinic appointment?
    - Probe participants to ascertain their reasons for agreeing or disagreeing with the above question.

16) Attendance and examination

- How well do the nurses attend to you in the consultation room?
    - Probe participants to ascertain their reasons for agreeing or disagreeing with the above question.
- Do the nurses do physical examination of your body in the consultation room?
    - Probe participants to ascertain their reasons for agreeing or disagreeing with the above question.

17) What would you like us to tell the National Department of Health concerning your individual needs, circumstances and challenges?

SUMMARY

- In order to be sure that we did not miss anything, the moderator will summarise the topics for the discussion.
- Is the summary accurate? Was anything left out?

QUESTIONS AND FEEDBACK

END OF THE FOCUS GROUP DISCUSSION

VUNENE STUDY – FOCUS GROUP DISCUSSION GUIDE

TOPIC GUIDE FOR CLINIC DEFAULTERS

1. **INTRODUCTION**
- Introduce members of the team
- The aim of this discussion is to understand patient experiences with the quality of care for chronic diseases in the primary health facilities and to know why the participants failed to come for clinic appointment for at least one month after the appointment given to them by the nurses
- We would like to get a very good understanding of your experiences in the clinics, some of which you may not have been able to discuss with us during interviews in the clinic last year.
- This discussion will contribute in understanding how services for chronic diseases are organized in the health facilities
- This study has the support of the Bushbuckridge Department of Health and Wits University

2. **ACTIVITIES**
- Eleven topics around quality of chronic disease care in the clinic will be discussed today
- Participation in this discussion will be free, fair, equal and in relaxed environment
- A report of this discussion will be sent to you
- There will be no mention of your names in the report or any other document(s)
- You will not be identified as a participant in this discussion
- Refreshments will be provided in the meetings
- You are free to leave the study at any time
- We would like to know if there are suggested modifications to this programme

3. **WRITTEN INFORMED CONSENT**

- Written informed consent, signed by participants, study investigator or senior qualitative field worker is obtained

INTRODUCTION

Chronic diseases are diseases (e.g. hypertension, diabetes and HIV) that require regular and on-going, usually for a long time contact with health facilities. Since June 2011, the National Department of Health has been testing the integrated chronic disease management (ICDM) model of care in all the clinics Bushbuckridge sub-district. The aim of this model is to improve health outcomes of patients with chronic diseases and improve service delivery in health facilities.

We invite you to describe your experiences with the quality of care in the clinics with respect to the topics below. **You are not expected to name the chronic disease for which you are being managed in the clinic.**

Topics for the discussion

1) General satisfaction with the quality of the integrated chronic disease care

- In general, could you tell us how satisfied or dissatisfied you are with the integrated chronic disease care in the health facility you attend?
    - Prompt participants to explain the reason for their satisfaction or dissatisfaction
- All of you have been receiving treatment in the clinics for at least two years. Have you noticed any changes in the way services for chronic diseases have been combined?
    - Prompt participants to discuss any positive or negative changes in the combined/integrated chronic disease services in the clinics since June 2011
- How perfect are these services?
    - Prompt participants to consider what works and does not work from their point of view and invite them to explain why they feel this way
    - Invite participants to describe specific examples

2) Technical quality of care and confidence in the nurses

- What are your views on the clinical skills of the nurses who treat chronic diseases?
    - Do you wonder if their diagnosis is correct
    - Do you have doubts about the clinical skills of the nurses
    - Invite participants to illustrate their opinions and describe specific instances

3) Interpersonal relations and friendliness

- Tell us whether the nurses are very friendly and polite to you
    - Invite participants to explain why they agree or disagree with the above question

4) Professionalism and competence of nurses

- What do you think about the professional conduct of the nurses?
    - Probe participants to narrate specific instances of professional misconduct of the nurses
- What do you think about the competences of the nurses to manage chronic diseases?
    - Probe participants to describe specific instances of competence or incompetence of the nurses

5) Communication

- Tell us whether the nurses explain to you reason(s) for doing physical examination or requesting laboratory tests
    - Invite participants to describe such experiences
- Do the nurses sometimes ignore what you tell them?
    - Probe participants to provide illustrations with specific instances

6) Financial aspect of accessing care

- Do have to pay fees to access services in the clinics?
    - Invite participants to illustrate specific instances when fees were paid

- Are you able to afford the cost of transportation to the clinic?
    - Invite participants to describe distances between their homes and the clinic, and how much they have to pay for transport

7) Waiting time before seeing nurses and time spent with nurses in the consultation room

- How long do you wait to see the nurses before they attend to you?
    - Probe participants to describe their experiences with waiting time
- Do the nurses hurry too much when they attend to you in the consultation room?
    - Probe participants to describe their experiences

8) Accessibility of the integrated chronic disease services

- How hard or easy is it for you to access services in the clinic?
    - Invite participates to describe their experiences
- Describe your experiences in having access to a doctor or a specialist

9) Coherence of the integrated chronic disease services

- How well combined are the HIV, hypertension and diabetes services
    - Invite participants to describe their experiences

10) Referral

- Tell us whether the nurses sometimes refer you to the doctor or hospital when necessary
    - Invite participants to describe their experiences with referral.
    - Probe about back-referral from the doctors to the nurses in instances where participants were referred to the doctors by the nurses
    - Tell us your experiences when you were transferred from one clinic to another

11) Defaulter-tracing

- Tell us why you failed to come for your clinic appointment for three or more consecutive clinic appointments
    - Explain to the participants that the period for discussion is from 2013 to 2014
    - Invite participants to explain why they defaulted.
    - Invite participants to describe their specific experiences when they defaulted.
    - Probe participants to ascertain if the community (volunteer) health workers visited their houses when they missed clinic appointments
    - Probe to ascertain if participants are referred to the clinics by the community (volunteer) health workers

12) Supply of critical medicines

- Can you tell us about regular supply of critical medicines (antiretroviral drugs, antihypertensive medicines and anti-diabetes medicines) for the treatment of chronic diseases in the clinics?
    - Invite participants to describe their experiences with drug stock-outs.
    - What was the response of the nurses in such instances? (E.g. do nurses order drugs from neighbouring clinics in such instances of drug stock-outs?)

13) Equipment

- Can you tell us about adequacy and functionality of equipment in the clinics?
  - Invite participants to share their experiences on availability of equipment in the clinics (e.g. BP machines. Glucometers, weighing scale etc.).
  - Probe participants to ascertain instances when equipment was needed to provide care, but was not available
  - Invite participants to describe the functionality of equipment in the clinics (e.g. BP machines. Glucometers, weighing scale etc.).
  - How do the nurses cope in such instances when the equipment are broken?

14) Appointment system

- Can you tell us about the clinic appointment system?
- How hard is it to get an appointment for medical care as soon as you need it?
  - Invite participants to describe their specific experiences
  - What are the intervals of clinic appointment?
  - How do the nurses prescribe medications to last the period of clinic appointment?

15) Prepacking of medicines

- Do the nurses pack your medicines the day before your clinic appointment?
  - Probe participants to ascertain their reasons for agreeing or disagreeing with the above question.

16) Attendance and examination

- How well do the nurses attend to you in the consultation room?
  - Probe participants to ascertain their reasons for agreeing or disagreeing with the above question.
- Do the nurses do physical examination of your body in the consultation room?
  - Probe participants to ascertain their reasons for agreeing or disagreeing with the above question.

17) What would you like us to tell the National Department of Health concerning your individual needs, circumstances and challenges?

SUMMARY

- In order to be sure that we did not miss anything, the moderator will summarise the topics for the discussion.
- Is the summary accurate? Was anything left out?

QUESTIONS AND FEEDBACK

THANK YOU FOR PARTICIPATING IN THIS DISCUSSION

END OF THE FOCUS GROUP DISCUSSION
