# Supplementary material for: Paradox of HIV stigma in an integrated chronic disease care in rural South Africa: Viewpoints of service users and providers
Source: PLoS One. 2020 Jul 31;15(7):e0236270. doi: 10.1371/journal.pone.0236270 (PMC7394420; doi:10.1371/journal.pone.0236270)
Supplement: S2 File — (PDF) [file pone.0236270.s002.pdf]

VUNENE STUDY – IN-DEPTH INTERVIEW GUIDE

IN-DEPTH INTERVIEW GUIDE FOR THE NURSE-IN-CHARGE OF A HEALTH FACILITY IN BUSHBUCKRIDGE SUB-DISTRICT

1. INTRODUCTION

- Introduce yourself

  The aim of this discussion is to get your perspective as the manager of your health facility concerning the quality of care in the integrated chronic disease management (ICDM) model

- This discussion will contribute in understanding how services for chronic diseases are organized in the health facilities
- This study has the support of the Bushbuckridge Department of Health and Wits University

2. ACTIVITIES

- Twelve topics around quality of chronic disease care in the clinic will be discussed today
- A report of this discussion will be sent to you
- There will be no mention of your names in the report or any other document(s)
- You are free to leave the study at any time
- We would like to know if there are suggested modifications to this programme

3. WRITTEN INFORMED CONSENT

- Written informed consent, signed by the facility manager and the study investigator, is obtained

INTRODUCTION

Chronic diseases are diseases (e.g. hypertension, diabetes and HIV) that require regular and on-going, usually for a long time contact with health facilities. Since June 2011, the National Department of Health has been testing the integrated chronic disease management (ICDM) model of care in all the clinics Bushbuckridge sub-district. The aim of this model is to improve

health outcomes of patients with chronic diseases and improve service delivery in health facilities.

We invite you to describe your experiences as a facility manager concerning the quality of care in the clinics with respect to the topics below.

Topics for discussion

1) General satisfaction with the quality of the integrated chronic disease care

- In general, could you talk about how satisfied or dissatisfied you are with the integrated chronic disease care in your health facility?
    - Prompt further to know the reason for her satisfaction or dissatisfaction
- Have you noticed any changes in the way services for chronic diseases have been combined since initiation of the integrated chronic disease care model?
    - Prompt to ascertain any positive or negative changes in the combined/integrated chronic disease services since June 2011
- How perfect are these services?
    - Prompt further to ascertain what works and what does not work and invite her to explain why she feels this way
    - Invite her to provide specific examples

2) Technical quality of care and confidence in the nurses

- What are your views on the clinical skills of the nurses who treat chronic diseases?
    - Do you wonder if their diagnosis is correct
    - Do you have doubts about the clinical skills of these nurses
    - Probe to illustrate her opinions and describe specific instances

3) Interpersonal relations and friendliness

- Are nurses in your health facility friendly and polite to patients?
    - Probe to know why she agrees or disagrees with the above question

4) Professionalism and competence of nurses

- What do you think about the professional conduct of the nurses?
    - Probe to ascertain specific instances of professional misconduct of the nurses
- What do you think about the competences of the nurses to manage chronic diseases?
    - Probe further for instances of competence or incompetence of the nurses

5) Communication

- Do nurses explain to patients reason(s) for doing physical examination or requesting laboratory tests
    - Probe further for such experiences
- Do the nurses sometimes ignore what patients tell them?
    - Probe further to know specific instances

6) Financial aspect of accessing care

- Do patients pay fees to access services in your health facility?

    - If yes, provide specific instances when fees were paid
- Are patients able to afford the cost of transportation to the clinic?
    - Invite her to describe patients' frustrations of having to pay for the cost of transportation from their homes to the clinic

7) Waiting time before seeing nurses and time spent with nurses in the consultation room

- How long do patients have to wait to see the nurses before being attended?
    - Probe to ascertain experiences with patient waiting time
- Do nurses spend quality time with patients in the consultation room?
    - Probe further to ascertain time patients spend with nurses during consultations

8) Accessibility of integrated chronic disease services

- How hard or easy is it for patients to access services in the clinic?
    - Probe to ascertain challenges patients have with accessing care
- Describe patients' experiences in having access to a doctor or a specialist

9) Coherence of the integrated chronic disease services

- How well integrated are the HIV, hypertension and diabetes services
    - Invite her to describe how well integrated HIV, hypertension and diabetes services are

10) Referral

- Do nurses sometimes refer patients to the doctor or hospital when necessary?
    - Invite her to describe experiences with patient referral.
    - Probe about back-referral from the doctors to the nurses in instances where patients were referred to the doctors by the nurses
    - What are your experiences when patients are transferred from one clinic to another?

11) Defaulter-tracing

- Do the community (volunteer) health workers visit patients when they miss three consecutive clinic appointments in the period from 2013 to 2014?
    - Invite her to explain why patients default.
    - Probe to ascertain if patients are referred back to the clinics by the community (volunteer) health workers

12) Supply of critical medicines

- Can we talk about regular supply of critical medicines (antiretroviral drugs, antihypertensive medicines and anti-diabetes medicines) for the treatment of chronic diseases in your health facility?
    - Invite her to describe situations with drug stock-outs.
    - How do you respond in such instances? (E.g. do you order for drugs from neighbouring clinics in such instances of drug stock-outs?)

13) Equipment

- How adequate and functional are the equipment in the clinics?

- Probe further on availability of equipment in the clinics (e.g. BP machines. Glucometers, weighing scale etc.).
- Probe further to ascertain instances when equipment was needed to provide care, but was not available
- Probe further on the functionality of equipment in the clinics (e.g. BP machines. Glucometers, weighing scale etc.).
- How do you deal with instances when equipment are broken?

14) Appointment system

- Can you talk about the clinic appointment system?
- How hard is it for patients to get an appointment for medical care as soon as it is need?
    - Probe further to describe specific experiences
    - What are the intervals of clinic appointment?
    - How do the nurses prescribe medications to last the period of clinic appointment?

15) Prepacking of medicines

- Do the nurses pack medicines the day before patients' clinic appointment?
    - Probe further to ascertain reasons for agreeing or disagreeing with the above question.

16) Attendance and examination

- How well do nurses attend to patients in the consultation room?
    - Probe further to ascertain whether or not nurses attend the patients.
- Do nurses conduct physical examination of patients in the consultation room?
    - Probe further to ascertain whether or not nurses examine patients in the consultation rooms.

17) What would you recommend to the Provincial and National Departments of Health?

SUMMARY

- In order to be sure that we did not miss anything, the interviewer will summarise the topics.

QUESTIONS AND FEEDBACK

THANK YOU FOR PARTICIPATING IN THIS INTERVIEW

END OF THE IN-DEPTH INTERVIEW
