# Supplementary material for: Paradox of HIV stigma in an integrated chronic disease care in rural South Africa: Viewpoints of service users and providers
Source: PLoS One. 2020 Jul 31;15(7):e0236270. doi: 10.1371/journal.pone.0236270 (PMC7394420; doi:10.1371/journal.pone.0236270)
Supplement: S3 File — (PDF) [file pone.0236270.s003.pdf]

Listed below are some things people say about health care. Please read each one carefully, keeping in mind the health care you are receiving now. (If you have not received care recently, think about what you would expect if you needed care today.) We are interested in your feelings, good and bad, about the health care you have received. The phrase "health care provider" includes physicians, physician assistants and nurse practitioners.

How strongly do you AGREE or DISAGREE with each of the following statements? (Circle One Number on Each Line)

	Strongly Agree	Agree	Uncertain	Disagree	Strongly Disagree
1. Health care providers are good about explaining the reason for medical tests (P1).					
2. I think my health care provider's office has everything needed to provide complete medical care (S1).[a]					
3. The health care I have been receiving is just about perfect (O1).[b]					
4. Sometimes health care providers make me wonder if their diagnosis is correct (O3).					
5. I feel confident that I can get the health care I need without being set back financially (D1).					
6. When I go for health care, the provider is careful to check everything when treating and examining me (P11).					
7. I have to pay for more of my health care than I can afford (D2).					
8. I have easy access to the medical specialists I need (P5 & P6).[c]					
9. Where I get health care, people have to wait too long for emergency treatment (O4).					
10. Health care providers act too businesslike and impersonal toward me (P3).[d]					
11. My health care provider treats me in a very friendly and courteous manner (P4).					
12. Those who provide my health care sometimes hurry too much when they treat me (P13).					
13. Health care providers sometimes ignore what I tell them (P2).					
14. I have some doubts about the ability of the health care providers who treat me (O5).					
15. Health care providers usually spend plenty of time with me (P12).					
16. I find it hard to get an appointment for health care right away (P14).					
17. I am dissatisfied with some things about the health care I receive (O2).[e]					
18. I am able to get health care whenever I need it (S4).					

[a]Rephrased to reflect availability of functional equipment for chronic disease care (structure - S1)
[b]Rephrased to reflect satisfaction with coherence of integrated chronic disease care (outcome - O1)
[c]Statement changed to reflect referral of patients to the hospitals (process - P5 and P6)
[d]Statement changed to reflect professional conduct of the nurses during clinical duties (process - P3)
[e]Rephrased to reflect dissatisfaction with coherence of integrated chronic disease care (outcome - O2)
